# T2-weighted imaging-based deep-learning method for noninvasive prostate cancer detection and Gleason grade prediction: a multicenter study

**DOI:** 10.1186/s13244-024-01682-z

**Published:** 2024-05-07

**Authors:** Liang Jin, Zhuo Yu, Feng Gao, Ming Li

**Affiliations:** 1 Radiology Department, Huashan Hospital, Affiliated with Fudan University, Shanghai, 200040 China; 2 Radiology Department, Huadong Hospital, Affiliated with Fudan University, Shanghai, 200040 China; 3 School of Information and Safety Engineering, Zhongnan University of Economics and Law, Wuhan, China; 4 Institute of Functional and Molecular Medical Imaging, Shanghai, 200040 China

**Keywords:** Prostate, Cancer, Magnetic resonance imaging, Radiologist, Gleason

## Abstract

**Objectives:**

To noninvasively detect prostate cancer and predict the Gleason grade using single-modality T2-weighted imaging with a deep-learning approach.

**Methods:**

Patients with prostate cancer, confirmed by histopathology, who underwent magnetic resonance imaging examinations at our hospital during September 2015–June 2022 were retrospectively included in an internal dataset. An external dataset from another medical center and a public challenge dataset were used for external validation. A deep-learning approach was designed for prostate cancer detection and Gleason grade prediction. The area under the curve (AUC) was calculated to compare the model performance.

**Results:**

For prostate cancer detection, the internal datasets comprised data from 195 healthy individuals (age: 57.27 ± 14.45 years) and 302 patients (age: 72.20 ± 8.34 years) diagnosed with prostate cancer. The AUC of our model for prostate cancer detection in the validation set (*n* = 96, 19.7%) was 0.918. For Gleason grade prediction, datasets comprising data from 283 of 302 patients with prostate cancer were used, with 227 (age: 72.06 ± 7.98 years) and 56 (age: 72.78 ± 9.49 years) patients being used for training and testing, respectively. The external and public challenge datasets comprised data from 48 (age: 72.19 ± 7.81 years) and 91 patients (unavailable information on age), respectively. The AUC of our model for Gleason grade prediction in the training set (*n* = 227) was 0.902, whereas those of the validation (*n* = 56), external validation (*n* = 48), and public challenge validation sets (*n* = 91) were 0.854, 0.776, and 0.838, respectively.

**Conclusion:**

Through multicenter dataset validation, our proposed deep-learning method could detect prostate cancer and predict the Gleason grade better than human experts.

**Critical relevance statement:**

Precise prostate cancer detection and Gleason grade prediction have great significance for clinical treatment and decision making.

**Key Points:**

- Prostate segmentation is easier to annotate than prostate cancer lesions for radiologists.
- Our deep-learning method detected prostate cancer and predicted the Gleason grade, outperforming human experts.
- Non-invasive Gleason grade prediction can reduce the number of unnecessary biopsies.

**Graphical Abstract:**

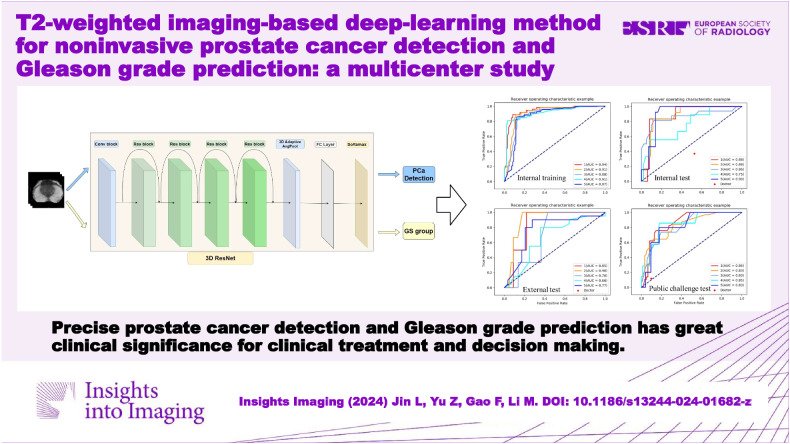

## Introduction

Prostate cancer (PCa) has gained considerable attention worldwide given that it is the most common cancer in male individuals in the Western world [1]. The 2022 European Association of Urology guidelines and 2019 UK National Institute for Health and Care Excellence guidelines suggest using multiparametric magnetic resonance imaging (MRI) for detecting early-stage PCa before biopsy [2]. However, assessments of PCa using MRI are susceptible to low inter-reader agreement (< 50%), suboptimal interpretation, and overdiagnosis [3, 4].

Pathological biopsy results are the gold standard for PCa classification. However, biopsy cores mostly refer to positive lesions (Prostate Imaging Reporting and Data System [PI-RADS v2] score ≥ 3), limiting the ability to detect all PCa lesions [5–7]. Furthermore, the annotations of biopsy-confirmed lesions highly depend on the interpretation of the radiologist [8].

Artificial intelligence (AI)-based approaches have been widely applied for the detection and classification of PCa tumors. Given the advantages of machine-learning or deep-learning approaches, most studies [9] have used multimodal imaging approaches, such as T2-weighted imaging (T2WI) combining diffusion-weighted imaging (DWI), the apparent diffusion coefficient (ADC), dynamic contrast-enhanced imaging (DCE-MRI), and DWI with the ADC. Multimodal imaging combines the strengths of various imaging modalities to provide comprehensive information. AI algorithms can utilize this diverse and rich data to enhance the accuracy and sensitivity of PCa detection and classification. Each imaging technique offers unique insights into the nature of the tumor. For example, DWI and ADC maps are valuable for evaluating tumor cell density, while DCE-MRI provides information on vascularity. AI can analyze these characteristics in-depth, resulting in improved tumor characterization and grading. However, managing and integrating different data types from multiple imaging modalities can be challenging. Ensuring compatibility and effective synthesis of this data for AI analysis poses a significant hurdle. AI models may develop biases based on their training data or struggle to generalize across diverse populations or imaging systems, potentially impacting their reliability and accuracy. For instance, a recent editorial suggested that ADC values are not readily available in software applications and ADC cut-off values are affected by hardware-related factors (e.g., sequence parameters, choice and number of *b* values, variation in field homogeneity, or coil selection among MRI systems) [10]. Another study reported that, according to their data, neither qualitative nor quantitative DCE assessment is required in prostate MRI [11]. Furthermore, most studies manually segment PCa lesions with the help of experienced radiologists, while the annotation of PCa lesions is challenged by MRI scan quality, which is affected by parameters such as slice thickness and field of view [12].

Hence, in this study, we attempted to decrease the variation arising from multimodality images, although such images contribute greatly to PCa diagnosis. We proposed a deep-learning model using single-modality (T2WI) images with prostate segmentation instead of lesion segmentation to improve the AI-based approach for noninvasive PCa detection and Gleason grade prediction, which has great clinical significance for clinical treatment and decision making.

## Patients and methods

### Study population

This retrospective study was conducted in accordance with the Declaration of Helsinki and approved by the Institutional Review Board of our hospital. The need for obtaining patient consent was waived due to the retrospective nature of the study.

**Fig. 1:**
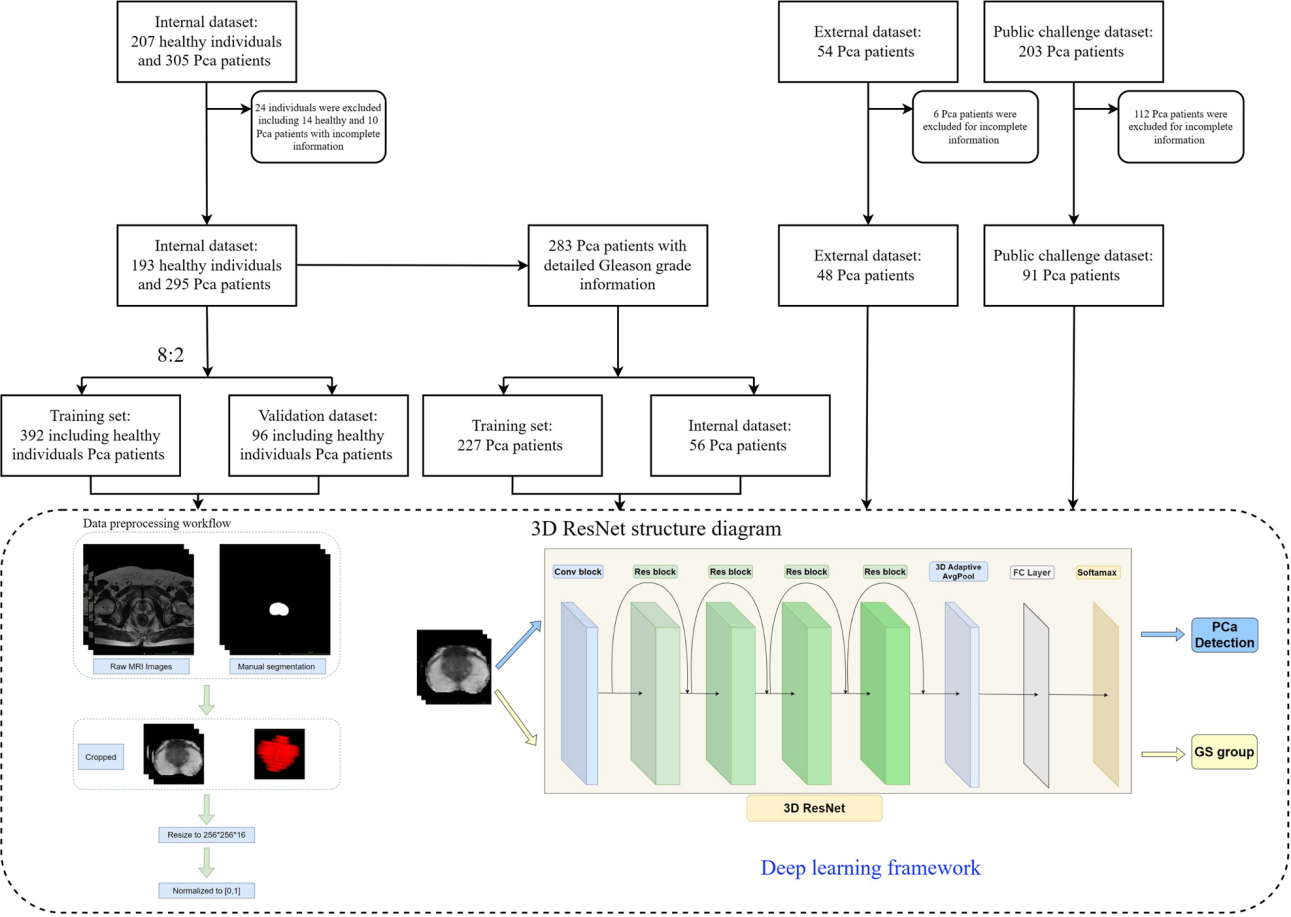
Flowchart of this study. The 3D ResNet structure diagram shows the following: The 3D ResNet utilizes three-dimensional (3D) convolutional layers, 3D pooling layers, and residual connections. The network begins with a 7 × 7 × 7 convolutional layer with 64 filters, a stride of 2, and padding of 3. Subsequently, a 2 × 2 × 2 max pooling layer with a stride of 2 is used for downsampling, halving the size of feature maps. The resulting network consists of four stages, each containing two residual blocks. Each residual block consists of two 3 × 3 × 3 convolutional layers. The first, second, third, and fourth stages have 128, 256, 512, and 1024 filters. Following all the residual blocks is a global average pooling layer. Finally, a fully connected layer with a number of nodes equal to the number of classes is used for classification, with a softmax activation function

Samples were collected from patients with PCa, confirmed by histopathology, who underwent MRI at our hospital between September 2015 and June 2022. The inclusion criteria were as follows: (1) MRI examination with T2WI and (2) no prostate biopsy, surgery, radiotherapy, or endocrine therapy performed before the MRI examination. Patients who had undergone catheter placement or previous treatment for PCa and those who exhibited artifacts on MRI were excluded (Fig. 1). External dataset 1 used for external validation was collected from another medical center. The inclusion criteria for the enrollment of external dataset 1 were the same as that applied to the internal dataset.

### Imaging acquisition and datasets

All T2WI images in both datasets were obtained using a 3-T MR system (MAGNETOM Skyra, Prisma, Vida, Siemens Healthcare, Erlangen, Germany) with a standard 18-channel phased-array body coil and a 32-channel integrated spine coil. Axial T2-weighted fast spin-echo imaging was performed with a slice thickness of 3–5 mm, no spacing, and a field of view of 12 × 12 cm^2^, including the entire prostate and seminal vesicles. The image acquisition parameters of external dataset 1 were required to be kept the same as our center. All internal MRI datasets were randomly divided into training and testing datasets. External datasets were collected from another medical center in China (external dataset 1) and public challenge datasets (public challenge validation) (https://prostatex.grand-challenge.org/).

### Prostate annotation

All internal datasets and external dataset 1 were manually annotated for the prostate region on T2WI by one radiologist with 5 years of experience in pelvic diagnosis; all annotations were confirmed by another radiologist with 10 years of experience in pelvic diagnosis. Manual annotation was performed using an open-source software (3D Slicer version 5.10; National Institutes of Health; https://www.slicer.org). All annotations were converted into the nii.gz format.

### Data preparation and preprocessing

Based on the annotation results, we cropped the original image and retained only the prostate area, thereby reducing the interference of nontarget areas on the model, and improving its efficiency. This step was performed using three-dimensional (3D)-connected domain technology. By calculating the mask, the minimum circumscribed 3D box of the prostate area could be obtained. To facilitate model training, the cropped image was resized to 256 × 256 × 16. Finally, all images were normalized to [0, 1] to reduce the differences between different datasets. The preprocessing is illustrated in Fig. 1.

The internal dataset was randomly divided into training (*n* = 228) and validation (*n* = 58) sets, at a ratio of 8:2. The external dataset 1 (*n* = 48) and public challenge dataset (*n* = 91) were used as external validation sets. Because of the unbalanced distribution of samples in the training set, we performed data expansion [13] for Category 1 (20 cases) with a small amount of data. The specific operation involved horizontally flipping the data of Category 1 (40 cases). After data expansion, the differences in classification and data volume in the training set were small (40:51:67:37:52), thereby improving the accuracy of the model in identifying all categories.

### Deep-learning framework

A 3D ResNet18 network, which is widely used in medical image analysis and has good performance, was used to construct the classification model [14, 15]. The process employed for the deep-learning model was a two-stage task: first, detecting PCas in the internal datasets, and second, predicting the Gleason grade in all PCas including the internal, external, and public challenge datasets. The 3D ResNet18 network consisted of a convolutional layer, four residual bolts, a three-dimensional adaptive average pool, a fully connected layer, and a softmax layer. The model used the Adam optimizer, with cross entropy as loss. A detailed explanation of the model is provided in Fig. 1. The input of the model was the cropped MRI images of size 256 × 256 × 16, whereas the output was the Gleason grade group classification result.

### Evaluation of the performance of deep learning

To better evaluate the performance of the model, we calculated the average precision, average recall, and F1 classification for the training, internal test, and external test sets, respectively. We also used the area under the curve (AUC) to evaluate the model performance. Concomitantly, we used a confusion matrix to visually demonstrate the classification ability of the model for all cases. When calculating each performance for the training set, we removed the cases in Category 1 that were duplicated owing to data augmentation.

In addition, we compared our model with two other deep-learning frameworks (3D ResNeXt [16] and 3D densenet [17]) for performance evaluation.

All experimental and statistical analyses were performed in a Linux environment (Ubuntu 20.04.2) with the following hardware conditions: an Intel CPU clocked at 3.70 GHz, 128 GB DDR4 memory, and an RTX3080 Ti graphics card with 10 GB memory. Python (version 3.9.7) from the Python Software Foundation was used as the programming language. We employed the PyTorch deep-learning framework (https://pytorch.org/) with key packages, such as SimpleITK (version 2.1.1), torch (version 1.11.0), torchvision (version 0.12.0), and scikit-learn (version 1.0.2).

### Radiologist interpretation for Gleason grade prediction

Reporting the Gleason grade using imaging is not a common task for radiologists; compared with the deep-learning model, the actual capability of radiologists to predict the Gleason grade is unknown. Hence, for Gleason grade prediction using T2WI, the senior radiologist with more than 10 years of experience in PCa diagnosis was blinded to all test sets of Gleason grade prediction, including the internal, external, and public challenge datasets.

## Results

The optimal parameters of the model were determined through multiple experiments: 200 training epochs, a batch size of 8, a learning rate of 0.0002, learning-rate updates every 20 epochs, a gamma of 0.8, and a dropout rate of 0.5.

### Performance of deep learning for PCa detection

For PCa detection, the internal MRI datasets consisted of 195 patients who were not diagnosed with PCa by radiologists with a follow-up period (age: 57.27 ± 14.45 years) and 302 who were diagnosed with PCa (age: 72.20 ± 8.34 years); no external validation was used as all patients were confirmed to have PCa based on histopathological results obtained through biopsy or surgery.

**Fig. 2:**
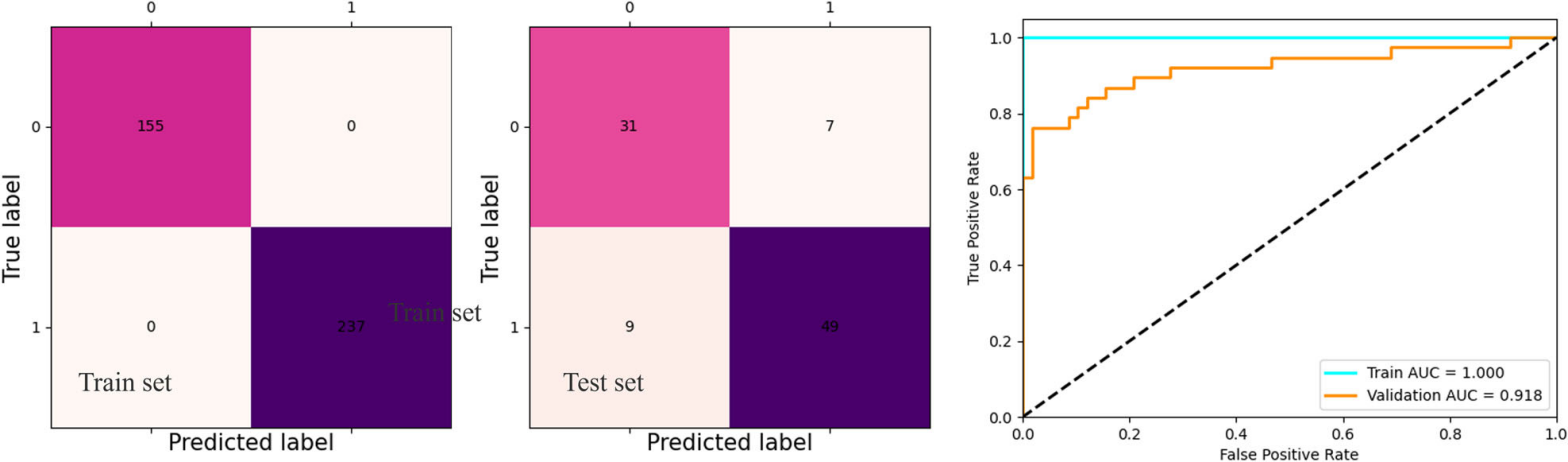
Performance of the deep-learning model for prostate cancer detection

The AUC of our model for PCa detection in the training set (*n* = 392, 80.3%) was 1, whereas that of the validation set (*n* = 96, 19.7%) was 0.918, as shown in Table 1 and Fig. 2.

**Table 1:** Performance of our deep-learning model and other models for PCa detection

Datasets and index		3D ResNeXt	3D Densenet	Our model
Training set (*n* = 392, 80.3%)	AUC	0.974	0.932	1.0
	Acc	0.989	0.955	1.0
	Recall	0.96	0.891	1.0
	F1	0.974	0.922	1.0
Validation set (*n* = 96, 19.7%)	AUC	0.911	0.853	0.918
	Acc	0.913	0.882	0.833
	Recall	0.907	0.851	0.875
	F1	0.91	0.866	0.86

*Acc* accuracy, *AUC* area under the curve, *PCa* prostate cancer

The AUCs of the PCa detection model built based on 3D ResNeXt and 3D densenet for the training sets were 0.932 and 0.974, respectively, and those for the test sets were 0.853 and 0.911, respectively. These results indicated that the model used in this study had satisfactory classification ability (Table 1).

### Performance of deep learning for Gleason grade prediction

For Gleason grade prediction, the data from 283 patients who were diagnosed with PCa, as confirmed by histopathological results obtained through biopsy or surgery were used from the internal dataset, of which data from 227 patients were used for training (age: 72.06 ± 7.98 years), whereas those from 56 patients were used for testing (age: 72.78 ± 9.49 years). The external and public challenge datasets consisted of data from 48 (age: 72.19 ± 7.81 years) and 91 (unavailable information on age) patients, respectively.

**Fig. 3:**
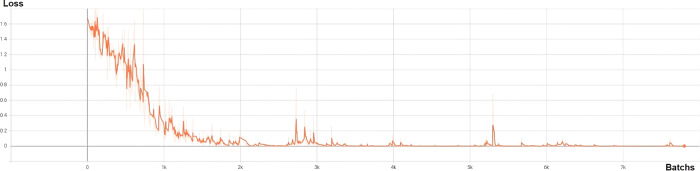
Performance of the deep-learning model for Gleason grade prediction

As shown in Fig. 3, during the training process, the loss of the model continued to decrease and stabilized after 3000 batches of training, indicating that the model achieved convergence. The AUC of our model for Gleason grade prediction for the training set (*n* = 227) was 0.902, whereas those for the validation (*n* = 56), external validation (*n* = 48), and public challenge validation (*n* = 91) sets were 0.854, 0.776, and 0.838, respectively. The details are presented in Tables 2–4.

**Table 2:** Patient characteristics

	Internal training and validation of patients with prostate cancer		External validation	Public challenge validation
Clinical diagnosis	Training	Validation	Validation	Validation
Number	227	56	48	91
Mean age (years)	72.06 ± 7.98	72.78 ± 9.49	72.19 ± 7.81	Unknown
Gleason grade	counts			
1	20	6	2	29
2	51	12	3	31
3	65	16	3	19
4	37	9	20	7
5	51	13	20	5

**Table 3:** Overall performance of our deep-learning model compared with that of other models for Gleason grade prediction

Datasets and index		3D ResNeXt	3D Densenet	Our model
Training set (*n* = 227)	AUC	0.857	0.838	0.902
	Acc	0.821	0.834	0.870
	Recall	0.842	0.787	0.861
	F1	0.831	0.810	0.865
Validation set (*n* = 56)	AUC	0.788	0.776	0.854
	Acc	0.785	0.768	0.857
	Recall	0.794	0.781	0.807
	F1	0.789	0.771	0.833
External validation set (*n* = 48)	AUC	0.698	0.703	0.776
	Acc	0.708	0.688	0.762
	Recall	0.604	0.646	0.677
	F1	0.642	0.661	0.718
Public challenge validation set (*n* = 91)	AUC	0.738	0.776	0.838
	Acc	0.735	0.768	0.806
	Recall	0.524	0.545	0.551
	F1	0.611	0.638	0.654

*Acc* accuracy, *AUC* area under the curve

**Table 4:** Performance of our deep-learning model for Gleason grade prediction

Datasets	AUC	Acc	Recall	F1
Training set (*n* = 227)				
Gleason grade group 1 (*n* = 20)	0.940	0.890	0.886	0.912
Gleason grade group 2 (*n* = 51)	0.907	0.883	0.886	0.907
Gleason grade group 3 (*n* = 65)	0.879	0.861	0.869	0.890
Gleason grade group 4 (*n* = 37)	0.914	0.861	0.807	0.882
Gleason grade group 5 (*n* = 51)	0.869	0.857	0.858	0.886
Validation set (*n* = 56)				
Gleason grade group 1 (*n* = 6)	0.880	0.893	0.833	0.625
Gleason grade group 2 (*n* = 12)	0.876	0.857	0.833	0.714
Gleason grade group 3 (*n* = 16)	0.863	0.821	0.813	0.722
Gleason grade group 4 (*n* = 9)	0.753	0.857	0.556	0.556
Gleason grade group 5 (*n* = 13)	0.901	0.857	1.000	0.765
External validation set (*n* = 48)				
Gleason grade group 1 (*n* = 2)	0.848	0.813	0.500	0.619
Gleason grade group 2 (*n* = 3)	0.900	0.875	0.667	0.757
Gleason grade group 3 (*n* = 3)	0.701	0.638	0.667	0.652
Gleason grade group 4 (*n* = 20)	0.663	0.708	0.800	0.751
Gleason grade group 5 (*n* = 20)	0.769	0.776	0.750	0.763
Public challenge validation set (*n* = 91)				
Gleason grade group 1 (*n* = 29)	0.859	0.758	0.414	0.535
Gleason grade group 2 (*n* = 31)	0.832	0.802	0.645	0.715
Gleason grade group 3 (*n* = 19)	0.828	0.824	0.526	0.642
Gleason grade group 4 (*n* = 7)	0.845	0.824	0.571	0.675
Gleason grade group 5 (*n* = 5)	0.827	0.823	0.600	0.694

*Acc* accuracy, *AUC* area under the curve

The AUCs of the Gleason grade prediction model built based on 3D ResNeXt and 3D densenet for the training sets were 0.857 and 0.838, those for the validation sets were 0.788 and 0.776, those for the external validation sets were 0.698 and 0.703, and those for the public challenge validation sets were 0.738 and 0.776, respectively. These results demonstrated that the model used in this study exhibited superior classification ability (Table 3).

### Radiologist interpretation

**Fig. 4:**
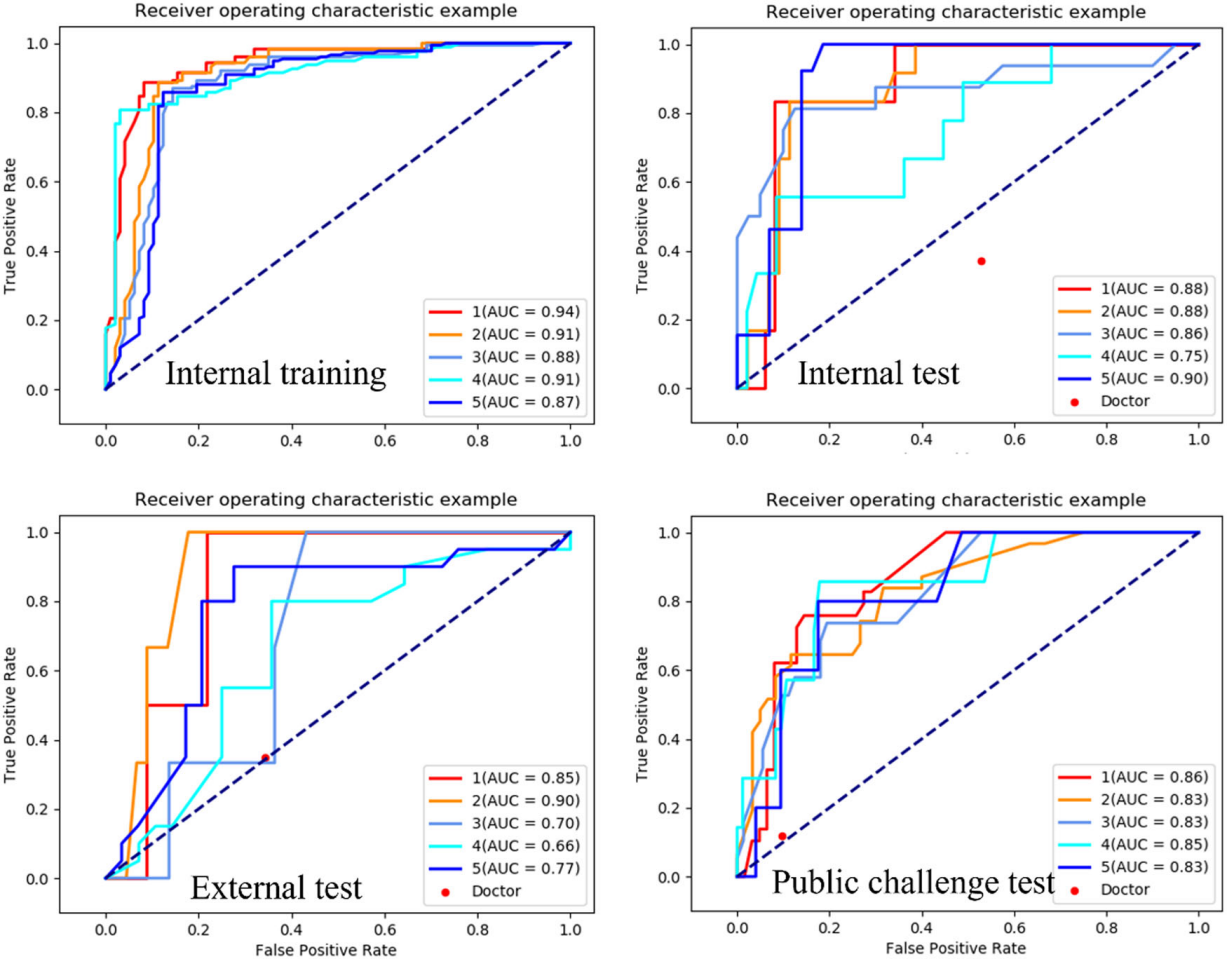
Performance of the deep-learning model and radiologist for Gleason grade prediction

The radiologist interpretations of Gleason grade prediction are shown in detail in Table 5 and Fig. 4.

**Table 5:** Radiologist performance for Gleason grade prediction

Datasets	Acc	Recall	F1
Validation set (*n* = 56)	0.528	0.528	0.528
Gleason grade group 1 (*n* = 6)	0.353	0.353	0.353
Gleason grade group 2 (*n* = 12)	0.308	0.308	0.308
Gleason grade group 3 (*n* = 16)	0.312	0.312	0.312
Gleason grade group 4 (*n* = 9)	1.000	1.000	1.000
Gleason grade group 5 (*n* = 13)	0.353	0.353	0.353
External validation set 1 (*n* = 48)	0.344	0.344	0.344
Gleason grade group 1 (*n* = 2)	0.000	0.000	0.000
Gleason grade group 2 (*n* = 3)	0.200	0.200	0.200
Gleason grade group 3 (*n* = 3)	0.091	0.091	0.091
Gleason grade group 4 (*n* = 20)	0.538	0.538	0.538
Gleason grade group 5 (*n* = 20)	0.889	0.889	0.889
Public challenge validation set (*n* = 91)	0.097	0.097	0.097
Gleason grade group 1 (*n* = 29)	0.111	0.111	0.111
Gleason grade group 2 (*n* = 31)	0.262	0.262	0.262
Gleason grade group 3 (*n* = 19)	0.111	0.111	0.111
Gleason grade group 4 (*n* = 7)	0.000	0.000	0.000
Gleason grade group 5 (*n* = 5)	0.000	0.000	0.000

*Acc* accuracy

## Discussion

Our proposed deep-learning model demonstrated good performance for PCa detection and Gleason grade prediction with T2WI, which was comparable to that reported in previous studies using multimodal imaging [9]. Although the PCa detection performance of our model did not show better performance compared with that of 3D ResNeXt, its Gleason grade prediction performance was the best among the three models tested. As a two-stage task in this study, the performance of 3D ResNeXt with the external validation set 1 and public challenge validation set was the worst among the three models, in contrast to our model that had the best performance. We noted that the performance of all models was more significantly decreased when used with the external validation set 1 than when used with the public challenge validation set. This was attributed to the image quality of T2WI images (e.g., field of view and slice thickness); the public challenge validation set is characterized by thinner slice thickness (https://prostatex.grand-challenge.org/) compared with that of the other two datasets, indicating that the better performance of the deep-learning model with the public challenge validation set compared with that when using the external validation set was influenced by image quality parameters. Furthermore, our model had better performance for Gleason grade prediction compared with that of radiologist interpretations using only T2W images.

Compared with Ishioka et al [18], who evaluated 335 patients using T2WI with a 0.8-mm slice thickness and achieved a model in two separate populations with AUCs of 0.636 and 0.645 for PCa detection, we used multicenter datasets and achieved an average AUC of 0.99 for PCa detection and an above average AUC of 0.87 for Gleason grade prediction. Compared with Schleb et al [19] (for a PI-RADS cut-off score of ≥ 3 vs. ≥ 4 on a per-patient basis), Arif et al [20] (for 292 patients with low-risk PCa), and Winkel et al [21] (for patients with lesions with PI-RADS score > 3 without histopathology validation), we demonstrated the good performance of our model in PCa detection and Gleason grade prediction for patients with all PCa grades. AI has become popular in recent years and has been heavily applied for PCa detection. Indeed, a meta-analysis [22] that included 12 studies reported an overall pooled AUC of 0.86, with a range of 0.81–0.91 in clinically significant PCa detection. As proposed in another brief summary of deep learning-based AI applications [12], most deep learning-based approaches are applied on naturally large-scale, diverse, and well-annotated datasets of both training and external validation testing datasets, whereas PCa lesion annotations are full of challenges even for experienced radiologists. Although the findings in this study were based on < 1000 case sample sizes, we tried using easy prostate annotation compared with PCa lesion annotation for wide validation across different medical centers in China, in addition to public challenge validation.

Multiparameter MRI shows high sensitivity and specific diagnostic capability for the clinical management of PCa by combining anatomical T1-weighted imaging, T2WI, functional DWI, DCE-MRI, and magnetic resonance spectroscopy [23]. As mentioned in a previous review of PCa imaging with a focus on deep-learning methods from 2014, Giannini et al [9] used T2WI and ADC for lesion detection, Matulewicz et al [24] used DCE for PCa classification, and Abraham et al [25] used T2WI, ADC, and DWI for PCa classification. These studies [9] used T2WI or ADC or both, DWI, and DCE-MRI for PCa classification; only Alkadi et al [26] used T2WI for PCa detection. Our model exhibited reliable performance using a large-scale multicenter dataset compared with the study by Alkadi et al, which had a very small sample size (19 patients). The strength of our study was that our deep learning model using single-modality imaging (T2WI) achieved good performance.

Prostate biopsy is an invasive procedure, and its benefits are evident when the diagnosis is accurate. Typically, a biopsy is recommended for lesions with a PI-RADS score of 4 or 5, indicating a high or very high likelihood of clinically significant cancer [27]. For lesions scored as PI-RADS 3, the decision to perform a biopsy may depend on other factors, such as PSA levels, PSA density, and the patient’s overall clinical context and risk factors [27–29]. Lesions with PI-RADS scores of 1 or 2 are generally considered low risk, and an immediate biopsy may not be necessary, although ongoing surveillance may be recommended. It is important to consider the individual circumstances, preferences, and overall health status of the patient when making the decision to perform a biopsy, in consultation with their healthcare provider.

One study presented at the RSNA 2020 highlighted the absence of specific guidelines for managing PI-RADS 3 patients, noting that a significant number of these patients could develop clinically significant PCa within a relatively short period. This suggests a potential need for a more proactive approach to their care management [30]. Another perspective mentioned in RSNA News pointed out that upgrading to PI-RADS category 3 may lead to unnecessary biopsies, underscoring the delicate balance between being overly cautious and avoiding unnecessary interventions [31]. Applied Radiology expanded on the management of PI-RADS 3 lesions, acknowledging the challenges posed by their overlapping findings with benign conditions. The article mentioned that the European Association of Urology guidelines recommend biopsy for all PI-RADS 3 lesions, emphasizing the importance of patient factors such as age, comorbidities, and treatment preferences in decision-making. The article also highlighted the utility of prostate-specific antigen density (PSAD) in assessing the risk of clinically significant cancer in PI-RADS 3 lesions, with higher PSAD levels indicating a greater likelihood of clinically significant PCa [32]. These insights indicate that while there is a consensus on the need for a thorough evaluation of PI-RADS 3 lesions, the approach to biopsy may vary based on factors such as patient-specific risk factors, additional imaging characteristics, and professional guidelines. The ongoing development of guidelines and the use of additional markers such as PSAD may help refine the decision-making process for these intermediate-risk lesions.

Therefore, if it were possible to accurately predict the staging of PCa non-invasively, it would effectively reduce unnecessary biopsies resulting from misdiagnosis and missed detections. Although our results showed better performance of the deep learning model on the public challenge dataset compared with the external validation dataset, we investigated the image quality factors such as slice thickness and field of view. We found that the public challenge dataset had a thinner slice thickness and provided a better display of the prostate with a wide field of view. This consistency is in line with the understanding that image quality can influence the performance of deep-learning models.

In this study, a senior radiologist with over 10 years of experience was invited to conduct the Gleason grade prediction. However, based on the results (Tables 3–5; Fig. 4), the performance of the deep-learning model (average AUC > 0.776) was significantly better than that of clinicians (average AUC < 0.5). Indeed, accurate prediction of the Gleason grade by clinicians is very difficult, as confidence in pathological histology diagnosis using only T2WI is lacking. In addition, as stated in a previous review [12], many reported performance metrics were mostly based on cross-validation, without including an actual radiologist vs. AI interaction; thus, these studies cannot represent a real-world setting. Our study demonstrated and compared the performance of both clinicians and models and although this does not fully reflect reality, it is still valuable.

Our study had some limitations. First, owing to the small sample size of the external and public challenge validation datasets, we were unable to show the performance of the model for PCa detection using external or public datasets; moreover, we did not accurately predict the Gleason score. Second, the pathological histology of some patients with PCa was obtained from biopsy, and some of these patients may have had cancer lesions of several Gleason grades. Therefore, we only predicted the highest Gleason grade instead of accurately predicting the Gleason grade of each cancerous lesion. Third, all MRI examinations were performed using 3 T devices, which means that the trained model may have limitations when applied to images from non-3T scans. Finally, our observation highlights a critical aspect of fairness and accuracy in comparing human and AI capabilities, particularly in specialized fields such as medical imaging and diagnosis. The Gleason score, which is used for grading the aggressiveness of PCa based on tissue samples, requires specific pathological expertise that radiologists might not typically possess. In contrast, the PI-RADS score is designed for radiologists to evaluate prostate MRI images and suggest the likelihood of clinically significant PCa, which aligns more with their training and expertise.

It is important to note that our intention in this study is not to highlight the lack of accuracy of physicians but rather to indicate that physicians may not be skilled at predicting Gleason grades. We aim for a more equitable and accurate comparison between AI and human performance in specialized tasks such as Gleason grade prediction. This approach not only ensures fairness but also contributes to a better understanding of how AI tools can complement professional expertise to improve diagnostic accuracy and patient care outcomes. We plan to address these limitations in future studies.

In conclusion, our deep-learning method using T2WI, validated through multicenter datasets, may provide a new approach to accurately diagnose PCa and predict Gleason grade. This has significant clinical significance for treatment and decision-making in clinical practice.

## Data Availability

The data presented in this study are available on request from the corresponding author. The data are not publicly available but may be retrieved with the permission of the corresponding author.

## Abbreviations

ADC: Apparent diffusion coefficient
AUC: Area under the curve
AI: Artificial intelligence
DWI: Diffusion-weighted imaging
DCE-MRI: Dynamic contrast-enhanced imaging
PCa: Prostate cancer
T2WI: T2-weighted imaging
